# Supplementary material for: Defining hierarchical protein interaction networks from spectral analysis of bacterial proteomes
Source: eLife. 2022 Aug 17;11:e74104. doi: 10.7554/eLife.74104 (PMC9427106; doi:10.7554/eLife.74104)
Supplement: Transparent reporting form [file elife-74104-transrepform1.docx]

***eLife’s* transparent reporting form**

We encourage authors to provide detailed information *within their submission* to facilitate the interpretation and replication of experiments. Authors can upload supporting documentation to indicate the use of appropriate reporting guidelines for health-related research (see [EQUATOR Network](http://www.equator-network.org/%20)), life science research (see the [BioSharing Information Resource](https://biosharing.org/)), or the [ARRIVE guidelines](http://www.plosbiology.org/article/info:doi/10.1371/journal.pbio.1000412) for reporting work involving animal research. Where applicable, authors should refer to any relevant reporting standards documents in this form.

If you have any questions, please consult our Journal Policies and/or contact us: [editorial@elifesciences.org](mailto:editorial@elifesciences.org).

**Sample-size estimation**

- You should state whether an appropriate sample size was computed when the study was being designed
- You should state the statistical method of sample size computation and any required assumptions
- If no explicit power analysis was used, you should describe how you decided what sample (replicate) size (number) to use

Please outline where this information can be found within the submission (e.g., sections or figure legends), or explain why this information doesn’t apply to your submission:

**Sample size for proteome analysis:** Explicated within the main text (first mention is line 67), Figure 1 caption, Methods.

**Sample size for biological benchmarks:** Explicated in Figure 3-source data 1, Figure 3-source data 1 (available for download from **github.com/arjunsraman/Zaydman_et_al**). All benchmarks are explicated in Methods; section titled ‘Assembling benchmarks described in **Figure 3A**’.

**Sample size for training RF models:** Explicated in Methods (line 657)

**Replicates**

- You should report how often each experiment was performed
- You should include a definition of biological versus technical replication
- The data obtained should be provided and sufficient information should be provided to indicate the number of independent biological and/or technical replicates
- If you encountered any outliers, you should describe how these were handled
- Criteria for exclusion/inclusion of data should be clearly stated
- High-throughput sequence data should be uploaded before submission, with a private link for reviewers provided (these are available from both GEO and ArrayExpress)

Please outline where this information can be found within the submission (e.g., sections or figure legends), or explain why this information doesn’t apply to your submission:

Information regarding replicates for experiments involving *Pseudomonas aeruginosa* can be found in Supplementary File 7 (available for download from **github.com/arjunsraman/Zaydman_et_al** as .zip file)

**Statistical reporting**

- Statistical analysis methods should be described and justified
- Raw data should be presented in figures whenever informative to do so (typically when N per group is less than 10)
- For each experiment, you should identify the statistical tests used, exact values of N, definitions of center, methods of multiple test correction, and dispersion and precision measures (e.g., mean, median, SD, SEM, confidence intervals; and, for the major substantive results, a measure of effect size (e.g., Pearson's r, Cohen's d)
- Report exact p-values wherever possible alongside the summary statistics and 95% confidence intervals. These should be reported for all key questions and not only when the p-value is less than 0.05.

Please outline where this information can be found within the submission (e.g., sections or figure legends), or explain why this information doesn’t apply to your submission:

Each p-value reported within the manuscript is followed by a description of the statistical test used to generate the p-value with the exception of p-values related to gene-set enrichment analyses.

p-values related to Gene Set Enrichment analyses are reported as output by DAVID analysis (v6.8), explicated in Methods (line 618). All p-values related to Gene Set Enrichment analyses generated by DAVID are found in Supplemenary Files 1 to 5 (available for download from **github.com/arjunsraman/Zaydman_et_al** as .zip file).

(For large datasets, or papers with a very large number of statistical tests, you may upload a single table file with tests, Ns, etc., with reference to sections in the manuscript.)

**Group allocation**

- Indicate how samples were allocated into experimental groups (in the case of clinical studies, please specify allocation to treatment method); if randomization was used, please also state if restricted randomization was applied
- Indicate if masking was used during group allocation, data collection and/or data analysis

Please outline where this information can be found within the submission (e.g., sections or figure legends), or explain why this information doesn’t apply to your submission:

No ‘group allocation’ was utilized in this manuscript.

**Additional data files (“source data”)**

- We encourage you to upload relevant additional data files, such as numerical data that are represented as a graph in a figure, or as a summary table
- Where provided, these should be in the most useful format, and they can be uploaded as “Source data” files linked to a main figure or table
- Include model definition files including the full list of parameters used
- Include code used for data analysis (e.g., R, MatLab)
- Avoid stating that data files are “available upon request”

Please indicate the figures or tables for which source data files have been provided:

Source data related to Figure 1A found in Figure 1-source data 1.

Source data related to Figure 3A found in Figure 3-source data 1; Figure 3-source data 2.

Source data related to Figure 6A found in Supplementary File 1.

Source data related to Figure 6—figure supplement 2 found in Supplementary File 2.

Source data related to Figure 6—figure supplement 3 found in Supplementary File 3.

Source data related to Figure 6—figure supplement 4 found in Supplementary File 4.

Source data related to Figure 7A,B; Figure 7—figure supplement 1 found in Supplementary File 5.

Source data related to Figure 7C found in Supplementary File 7.

Source data related to Figure 8; Figure 8—figure supplement 3 found in Figure 8-source data 1.

All tables are available for download as .zip files from **github.com/arjunsraman/Zaydman_et_al.**

All code used in the manuscript was written in Matlab v2020a; code for generating figures in the manuscript is available for download as .m scripts and .mat workspaces at **github.com/arjunsraman/Zaydman_et_al**. Instructions are in the Readme.m file included in the github repository.
